# Supplementary material for: Identification of Risk Loci for Radiotoxicity in Prostate Cancer by Comprehensive Genotyping of TGFB1 and TGFBR1
Source: Cancers (Basel). 2021 Nov 8;13(21):5585. doi: 10.3390/cancers13215585 (PMC8582951; doi:10.3390/cancers13215585)
Supplement: Supplementary file 1 [file cancers-13-05585-s001.zip › cancers-1423899-supplementary.pdf]

Supplementary Materials: Identification of Risk Loci for Radio-toxicity in Prostate Cancer by Comprehensive Genotyping of TGFB1 and TGFBR1

Manuel Guhlich, Laura Hubert, Caroline Patricia Nadine Mergler, Margret Rave-Fraenk, Leif Hendrik Dröge, Martin Leu, Heinz Schmidberger, Stefan Rieken, Andrea Hille and Markus Anton Schirmer

Supplementary Material

1. Supplementary Methods
2. Supplementary Tables: PCR primers, chemicals, kits, buffers, cell culture medium
3. Supplementary Figures

1. Supplementary Methods

Selection of genetic polymorphisms for genotyping

Data on genetic polymorphisms (single nucleotide and insertion/deletion) at the *TGFB1* and *TGFBR1* sites have been downloaded from the 1000 human genome database (www.internationalgenome.org/, integrated phase 1_version 3 as accessed by January 2014 and based on genome assembly GRCh37.p13. Five Caucasian populations were considered comprising a total number of 379 individuals: CEU, Utah residents of north-western European descent, N=85; FIN, Finnish in Finland, N=93; GBR, British in England and Scotland, N=89; IBS, Iberian Population in Spain, N=14; TSI, Toscani in Italia, N=98. The combined set of these data were analyzed and visualized by HaploView software, version 4.2 (www.broadinstitute.org/haploview/haploview) separately for the *TGFB1* and the *TGFBR1* genetic locus (see Supplementary Figures 1 and 2 below). The considered loci for genotyping comprised the entire region of these two genes with additional 5 kb at the flanking 5'- and 3'-sites (core region). If there was high linkage disequilibrium (LD, dark coloring in Supplementary Figures 1 and 2) of genetic polymorphisms located within with such beyond the core

region the bioinformatics analysis was extended to regions until there was no high LD anymore (i.e. $r^2<0.5$ with polymorphisms within the core region). Therefore, for *TGFB1* the 5'-upstream region was extended to 18 kb upstream and for *TGFBR1* to 6 and 10 kb upstream and downstream, respectively. Within these areas (in total 51 and 75 kb for *TGFB1* and *TGFR1*) genetic polymorphisms with a minor allele frequency (MAF) of at least 5% in the denoted five Caucasian populations were considered. Using the tagger algorithm implemented in the HaploView software a set of genetic polymorphisms covering the entire genetic variability with a MAF ≥ 5% in the defined areas at a pairwise stringency of $r^2=0.80$ was determined. This means if two polymorphisms show a pairwise LD of $r^2\geq0.80$ they were regarded as highly redundant and thus only one of them was selected for genotyping. Given these criteria, the bioinformatics analyses revealed 29 and 14 polymorphisms capturing the genetic diversity in the *TGFB1* and the *TGFBR1* area, respectively. The actually genotyped polymorphisms were 27 and 13 (see below).

Genotyping

Genomic DNA (gDNA) from whole EDTA-anticoagulated blood was isolated using the EZ1 DNA Blood 350 µl Kit (Qiagen, Hilden, Germany) on a BioRobot® EZ 1 system (Qiagen). The genetic polymorphisms selected according to the above-mentioned bioinformatic procedures were analyzed via multiplex primer extension method. The genetic sites of interest were first amplified by PCR according to supplier's instructions (Multiplex PCR Kit, Qiagen). PCR was started with an initial activation step at 95.0°C for 15 min. Then 40 cycles were run each with annealing at 65.0°C for 30 s and elongation at 72°C at 90 s. The PCR was finalized by a single elongation step at 72°C for 10 min. PCR primer sequences are provided in Supplementary Table S1 below. The samples were purified from excessive primers and dNTPs of the PCR by *exonuclease I* (8 units per sample) and *shrimp alkaline phosphatase* (SAP, 0.8 units) for 3 h at 37°C. Afterwards, the enzymes were inactivated at 80°C for 15 min. Then, specific primers binding next to the polymorphic site to be assessed were added and elongated by one fluorescence-labeled nucleotide (SNaPshot™, Thermo Fisher Scientific, Waltham, MA,

USA). Following a second purification with 0.5 units SAP the samples were boiled in 10 µl Hi-Di™ formamide supplemented with a size standard (GeneScan™ LIZ® 120 Thermo Fisher). The elongated primers were then analyzed in a 16-capillary sequencer (3100 Genetic Analyzer, formerly Applied Biosystems, now Thermo Fisher).

For one of the 29 *TGFB1* area polymorphisms selected due to bioinformatic analyses, rs201415320 (14,492 and 15,374 bp upstream of the transcription and translation start site of the single validated *TGFB1* mRNA isoform) the approached multiplex genotyping assay failed with several attempts performed. Another polymorphism of this area, rs2873369 (7892 and 8774 bp upstream of *TGFB1* transcription and translation start), is located within a 292 bp deletion. This deletion is covered by the successfully assayed marker rs2241713 at r^2=0.97. Thus, genotyping of this 292 bp deletion with pertinent rs2873369 was spared. One out of the 14 polymorphisms of the *TGFBR1* area (i.e. rs190034323) could not be verified in our analyses despite no hints for technical problems and was later on corrected in the 1000 human genome database to be monomorphic in Caucasians. The 27 and 13 genetic polymorphisms related to the *TGFB1* and *TGFBR1* area determined in the clinical cohort of 240 patients (full data set presented in Supplementary Data Excel sheet).

2. Supplementary Tables

Table S1: Primer sequences used for PCR amplification of the regions of interest.

Polymorphism	PCR forward primer (5'->3')	PCR reverse primer (5'->3')
**TGFB1**		
rs73047224	TCCTGGGCTAAACCAATCCTCCTATC	GGCACAACAAGGGCTGAGATTTG
rs8108357	"	"
rs75041078	CGCACACCACCAAGTCCAGCTA	GAGCCACTGAGACCACCCTCTCTC
rs8103493	CCTGGTGGTTCATGGCTGTCTG	TGTGCCCAGCCCCATATTCTATTG
rs2241713	ATGCCCCACTTGCAGAAGAGGCTAC	CAGCAACAACCACGGAAACCAAGAC
rs199538573	GCTGAAGTGGGTGGCCTGTTTG	CCCACACCCTGTCCACATGAGTC
rs1800468	GGACCCAGAACGGAAGGAGAGTCAG	TGCTGCATGGGGACACCATCTACA
rs1800469	"	"
rs1800470	CCCCACCACACCAGCCCTGTT	GCTTCACCAGCTCCATGTCGATAGTCTTG
rs1800471	"	"
rs6508976	AGCTGACAGCTCTGGGGTGGAGTC	AGGGGGAGAAACCACTTCCTCAAGGT
rs8108632	"	"
rs2241717	TCGGCTGGTTACAAGGTCCACCTAG	CCAGCTTGGCAACAGAGTGAGACAC
rs76206979	GCTGTTGGCACTCAAGACCTCAATTTG	GGCTGGGATTACGGCTATCCAGGTAC
rs73045292	CCAGTATTGCATCCCAACATTCCAAAG	GGCTGGGATTACGGCTATCCAGGTAC
rs200419705	GCTGTTGGCACTCAAGACCTCAATTTG	TTTGGTCCTGTCTCTGGGCTTTGTTAC
rs11466338	"	"
rs2278422	GCTGTTGGCACTCAAGACCTCAATTTG	TTTGGTCCTGTCTCTGGGCTTTGTTAC
rs11466344	GGGACCCAATGTTGCCTAGGAAGTC	CACACACCACCCAGCCAGCTAGT
rs6508975	CATGGGTCCTCTTACAAAATTCACCACTTAC	CCCCATGCAACCAAAAATTCACATACAAG
rs11466345	CAAACCCATGTCCAAGGGTCAGTCT	CCTGCCTCAGTCTCCCAAGTAGCTAAG
rs11466347	CCCAGCTAGGGTTGAACTGCATATTAAAG	CTGAACTCACAAAAGGGGAAGAACTAGTGTC
rs11466353	CCAGAAGAAGGCAGGCAGAGAGTAATAG	ATGTCCTTCACTCAGGGACCCTTAACTC
rs8179181	GAGGAGGGGCATGTGGCTTCT	TGCGTGTCCAGGCTCCAAATGTAG
rs7254679	CTCCCCCCACGACTTCCCTCT	GCCCCGTCGGGACAAGTCTG
rs73045282	CTAACACGTGCTGGGGACACACA	CAGTGTGGATTCGGATTTGTGTGTTC
rs10417924	CTAACACGTGCTGGGGACACACA	CAGTGTGGATTCGGATTTGTGTGTTC

Polymorphism	PCR forward primer (5'->3')	PCR reverse primer (5'->3')

**TGFBR1**		
rs149234874	TGGTGCTTAAAGCCCGAATTCATGT	CCCCACCCTCAATCACAAAGATTTC
rs72739266	"	"
rs10819635	GGTACAAGGAGCCTGGGAAATTGAC	CCAGCAAGTCAGCTGCAGAGCTA
rs10733708	GCCTCAGTGTTAGCAACTAAAATGCAGTGA	GCAAATGCCACTACTCCTGGGAGTTTAC
rs6478974	CTTGGCTGGAGAGGATGGCATGTG	TCCCTCTGCGGCACAATTAGACATTC
rs10512263	GTCTGGGATTCTACAGTGGAATTTTGTC	TTCGTAGGCCACCAAAGGTAAACTTC
rs10988716	CGGAAGGAGGTGTCTTCGTTTACG	TTCACCACTTGAAGTCAGTCTTGATTG
rs11568785	TGTGGGATTTCAGATGCCAGTATATCTC	GGAGCAGTTGTTGCCTAGGGCTAAG
rs10733710	CCTGCTCTGAAGCCAGAGTTACCTGAC	GCAGGGCTAGCTACTGCACAGTATTTG
rs11386616	ACAAATGCCCTGGCTTAAAGCAACAC	ACCCCTGGCCAACCTGTCTTACAAC
rs34733091	TGCCGAGAGAAATCAAAGGATCTAAATGAG	GGGTAAGGCAATTCCTCCAATTTTGTG
rs3983364	AACTCGCCTTTGCCAGTTTTTGTTG	CTGAGCCTTGGGGTTGATTCAAGTTAG
rs78471739	CCCAGATTTCAGGTCTGTGCAGATCTC	CCACATTTTTAGCTGGCGTAGGAGATATTG

Table S2: Chemicals and kits

Chemical/kit	Provider
5-FU (5-Fluoruracil)	Medac, Hamburg, Germany
Anti-phospho-Histone H2A.X (Ser139), FITC-markiert, Klon JBW 301	Merck Chemicals, Darmstadt, Germany
BSA	Sigma-Aldrich Chemie, Taufkirchen, Germany
CountBright™ Absolute Counting Beads	Thermo Fisher Scientific, Waltham, MA, USA
Exonuclease 1	Thermo Fisher Scientific, Waltham, MA, USA
EZ1 DNA Blood 350 µl Kit	Qiagen, Hilden, Germany
FCS (fetal calf serum)	Thermo Fisher Scientific, Waltham, MA, USA
GeneScan™ LIZ® 120	Thermo Fisher Scientific, Waltham, MA, USA
herring sperm DNA	Sigma-Aldrich Chemie, Taufkirchen, Germany
Hi-Di™ formamide	Thermo Fisher Scientific, Waltham, MA, USA
mouse serum	Thermo Fisher Scientific, Waltham, MA, USA
Multiplex PCR Kit	Qiagen, Hilden, Germany
Natriumazid (NaN3)	AppliChem, Darmstadt, Germany
Natriumfluorid (NaF)	AppliChem, Darmstadt, Germany
Natriummetavanadat	Sigma-Aldrich Chemie, Taufkirchen, Germany
Natriummolybdat (Na2MoO4)	Sigma-Aldrich Chemie, Taufkirchen, Germany
PBS Pulver (Dulbeccos 10-fold)	AppliChem, Darmstadt, Germany
Penicilline / Streptomycine	Thermo Fisher Scientific, Waltham, MA, USA
Propidiumiodid	Becton Dickinson, Franklin Lakes, NJ, USA
RNase A (70 %)	AppliChem, Darmstadt, Germany
RPMI-1640 medium	Thermo Fisher Scientific, Waltham, MA, USA
Recombinant human TGFβ1	PromoCell, Heidelberg, Germany
Shrimp alkaline phosphatase	Thermo Fisher Scientific, Waltham, MA, USA
SNaPshot™	Thermo Fisher Scientific, Waltham, MA, USA
Triton X-100	Carl Roth KG, Karlsruhe, Germany
Tris 100 %	Carl Roth KG, Karlsruhe, Germany
Vibrant® DyeCycle™ Ruby Stain	Thermo Fisher Scientific, Waltham, MA, USA

Table S3: Buffers and cell culture medium

Buffer/medium	Ingredients
Block-9 staining buffer	PBS with
	1 g/l BSA
	8 % mouse serum
	0.1 g/l RNase A
	10 mM NaF
	1 mM Na2MoO4
	1 mM NaVO3
	0.25 g / l herring sperm DNA
	0.1 % Triton X-100
	5 mM EDTA
	0.05 % NaN3
Cell culture medium	RPMI-Medium with
	15 % FCS
	1 % Penicilline/Streptomycine
PBS (pH 7.4)	128.5 mM NaCl
	2.8 mM KCl
	8.1 mM Na2PO4
	1.5 mM KH2PO4
Suspension Buffer	PBS mit
	1 g/l BSA
TE-Puffer (pH 7.5)	10 mM Tris
	1 mM EDTA

Table S4: Distribution of rectum, bladder, and combined toxicities

		Toxicity grade, No. (%)			
		**0**	**1**	**2**	**3**
Acute (*n*=240)	Rectum	65 (27.1)	119 (49.6)	55 (22.9)	1 (0.4)
	Urinary bladder	79 (32.9)	134 (55.8)	26 (10.9)	1 (0.4)
	Combined	30 (12.5)	139 (57.9)	69 (28.8)	2 (0.8)
Late (*n*=238)	Rectum	162 (68.1)	40 (16.8)	32 (13.4)	4 (1.7)
	Urinary bladder	168 (70.6)	56 (23.5)	9 (3.8)	5 (2.1)
	Combined	130 (54.1)	62 (25.8)	38 (15.8)	8 (3.3)

Acute toxicity was scored according to CTCAE, version 3.0, and late by LENT-SOMA, version 3.0.

Abbreviations: CTCAE = Common Toxicity Criteria assessment for Adverse Events; LENT-SOMA = Late Effects of Normal Tissues - Subjective, Objective, Management criteria with Analytic laboratory and imaging procedures.

3. Supplementary Figures

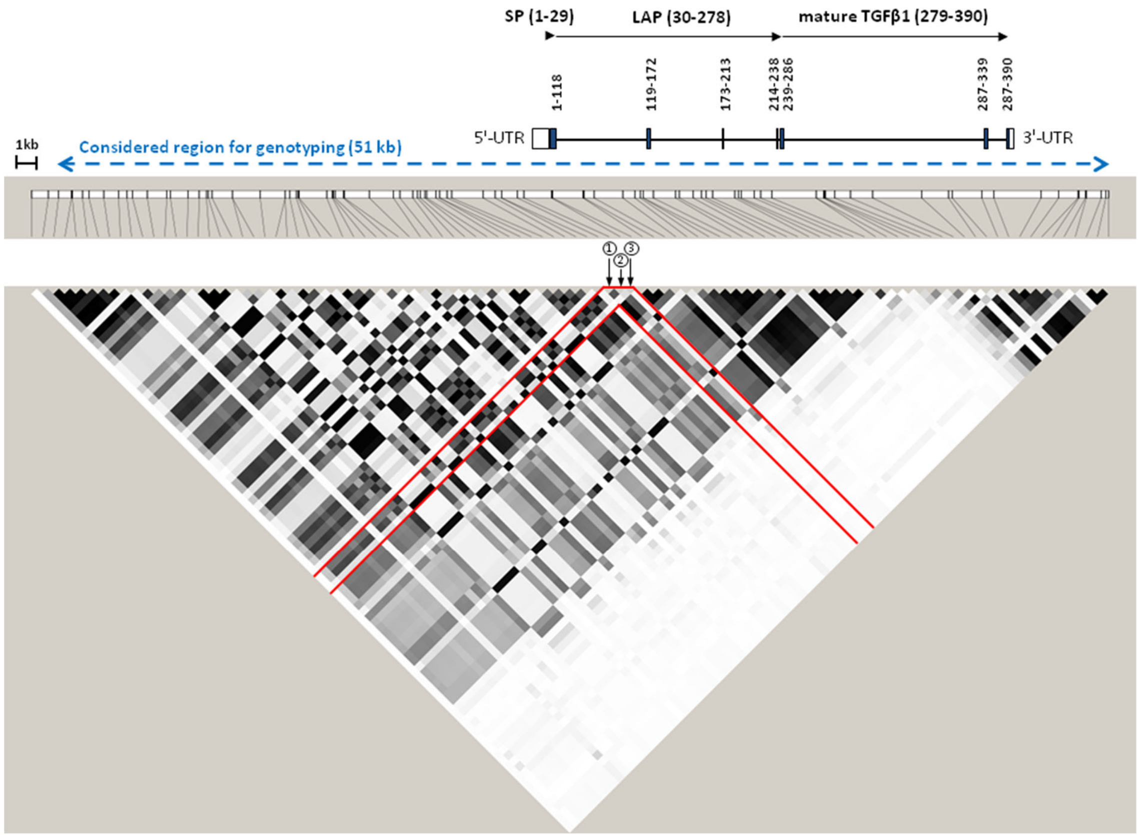

Figure S1. Plot of pairwise linkage disequilibrium at the *TGFB1* genetic region constructed from data based on Caucasian populations as described in the Methods section, chapter 2.4. SP = signal peptide, 1kb = 1,000 base pairs; three polymorphisms most investigated in literature are particularly denoted: ① = -509C>T (rs1800469), ② = 869T>C (rs1800470), ③ = 915G>C (rs1800471).

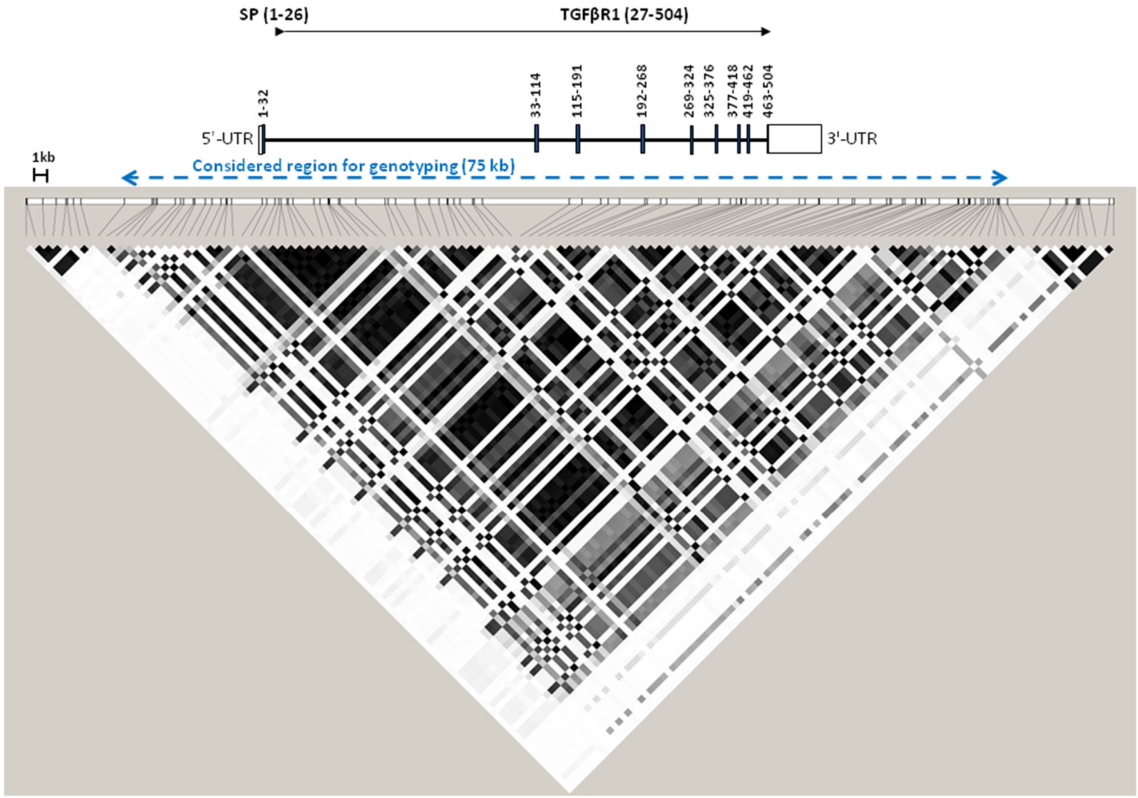

Figure S2. Pairwise linkage disequilibrium at the *TGFBR1* genetic region based on the same populations used for Supplementary Figure 1.

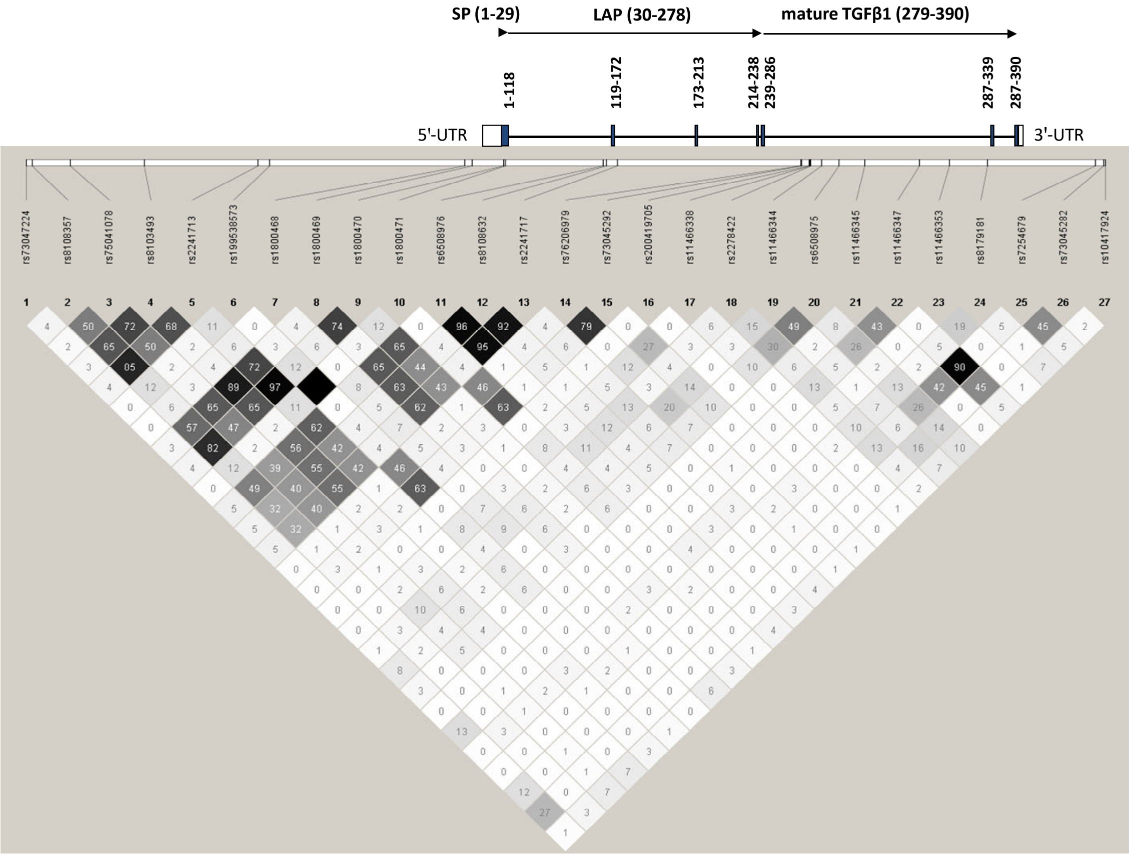

Figure S3. Pairwise linkage disequilibrium of the 27 *TGFB1* polymorphisms analyzed in the study population.

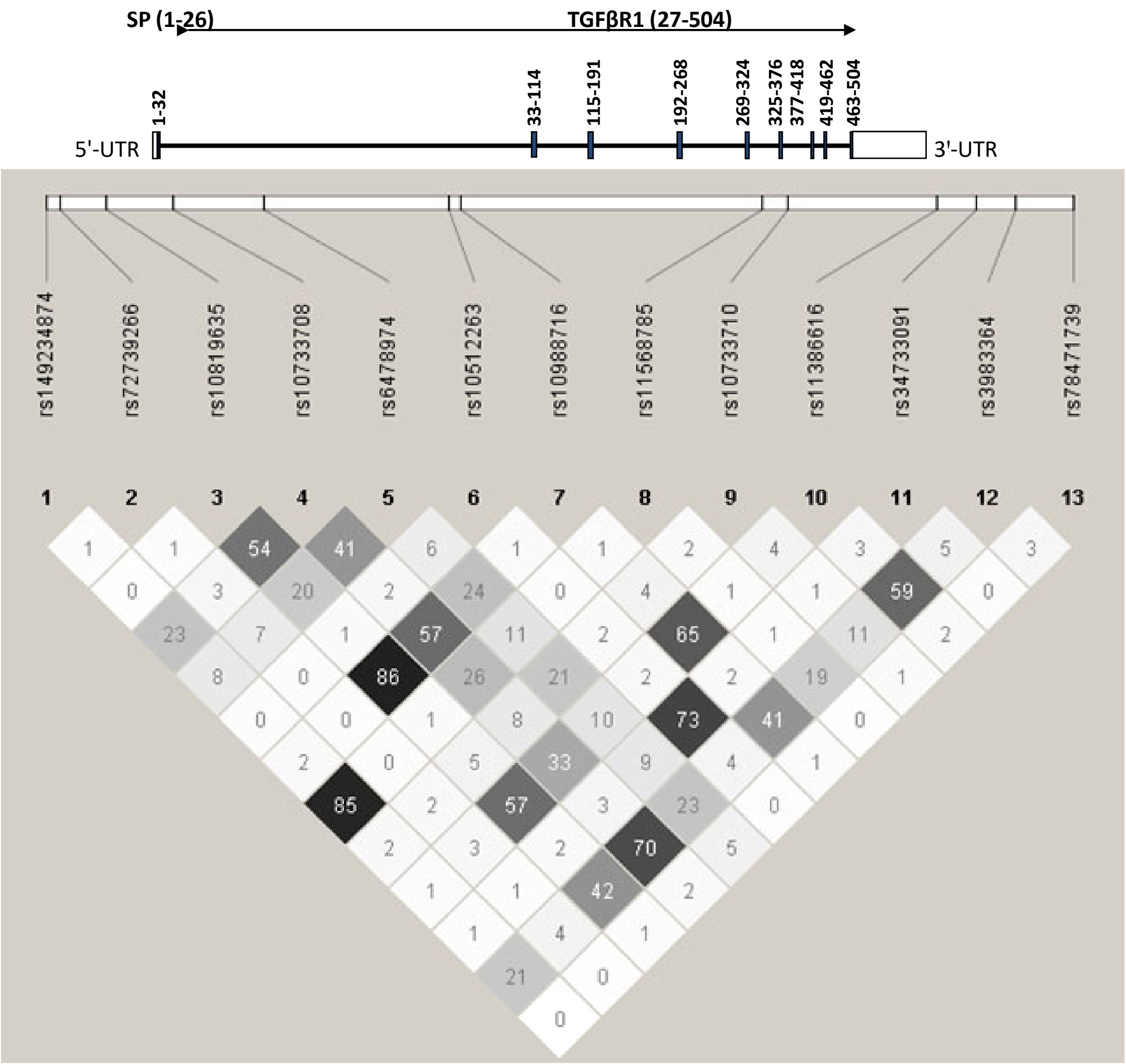

Figure S4. Pairwise linkage disequilibrium of the 13 *TGFBR1* polymorphisms analyzed in the study population.
